# Protective Carbon Overlayers from 2,3-Naphthalenediol Pyrolysis on Mesoporous SiO_2_ and Al_2_O_3_ Analyzed by Solid-State NMR

**DOI:** 10.3390/ma11060980

**Published:** 2018-06-09

**Authors:** Pu Duan, Xiaoyan Cao, Hien Pham, Abhaya Datye, Klaus Schmidt-Rohr

**Affiliations:** 1 Department of Chemistry, Brandeis University, Waltham, MA 02453, USA; pduan91@brandeis.edu (P.D.); xcao15@brandeis.edu (X.C.); 2 Department of Chemical & Biological Engineering and Center for Microengineered Materials, University of New Mexico, Albuquerque, NM 87131, USA; hipham@unm.edu (H.P.); datye@unm.edu (A.D.)

**Keywords:** NMR of conductive materials, carbon-coated mesoporous oxides, structure of carbon overlayers

## Abstract

Hydrothermally stable carbon overlayers can protect mesoporous oxides (SiO_2_ and Al_2_O_3_) from hydrolysis during aqueous-phase catalysis. Overlayers made at 800 °C by pyrolysis of 2,3-naphthalenediol deposited out of acetone solution were analyzed by solid-state ^13^C nuclear magnetic resonance (NMR) spectroscopy. Power absorption due to sample conductivity was prevented by diluting the sample in nonconductive and background-free tricalcium phosphate. While pyrolysis on SiO_2_ produced a predominantly aromatic carbon film, at least 15% of nonaromatic carbon (sp^3^-hybridized C as well as C=O) was observed on γ-Al_2_O_3_. These species were not derived from residual solvent, according to spectra of the same material treated at 400 °C. The sp^3^-hybridized C exhibited weak couplings to hydrogen, short spin-lattice relaxation times, and unusually large shift anisotropies, which are characteristics of tetrahedral carbon with high concentrations of unpaired electrons. Moderate heat treatment at 400 °C on SiO_2_ and Al_2_O_3_ resulted in yellow-brown and nearly black samples, respectively, but the darker color on Al_2_O_3_ did not correspond to more extensive carbonization. Aromatic carbon bonded to hydrogen remained predominant and the peaks of naphthalenediol were still recognizable; however, some of the chemical shifts differed by up to 5 ppm, indicating significant differences in local structure. On SiO_2_, additional sharp peaks were detected and attributed to 1/3 of the 2,3-naphthalene molecules undergoing fast, nearly isotropic motions.

## 1. Introduction

The transformation of biomass-derived feedstocks to valuable fuels and chemicals requires stable heterogeneous catalysts and catalyst supports. Since biomass feedstocks are oxygenated and soluble as well as reactive in water, the reactions are carried out in aqueous phase, often at temperatures of 473 K or higher [1,2,3,4,5]. Catalytically active metals are conventionally supported on mesoporous oxides such as alumina (Al_2_O_3_) and silica (SiO_2_). Designed for gas-phase reactions, these mesoporous oxides are not suitable for aqueous-phase reactions at elevated temperatures because they lose both surface area and structural integrity [6,7,8,9]. Previously, we reported a simple and inexpensive carbon-coating approach for modifying the surfaces of mesoporous oxide supports that made them hydrothermally stable in liquid water at 473 K [7]. Recently, we also reported on the benefits of adding thin carbon layers as overcoats to oxide-supported precious and nonprecious metal catalysts [10]. Using our carbon-overcoating approach, we showed that the catalysts were more active and stable for aqueous-phase reactions, such as furfural hydrogenation, than their uncoated counterparts due to a significant reduction in metal sintering as well as reduced loss of surface area and structural integrity of the oxide supports [10].

Among the techniques used to study carbon structures in catalytic materials, solid-state NMR is arguably the best available method for a comprehensive and quantitative characterization [11,12,13] of the composition of the carbon overlayers [14]. Raman spectroscopy has been much more widely used, but Raman spectra of carbon materials have repeatedly been misinterpreted. For instance, a peak at ~1580 cm^−1^ has been incorrectly assigned to graphitic C, even in materials containing >30 atom% oxygen and predominantly furan rings, which are clearly not graphitic [11,12,13]. In nanodiamond, an OH-bending Raman band [15] at ~1640 cm^−1^ that disappeared upon heating has long been misassigned to graphitic carbon [16]. Unlike NMR, which is intrinsically quantitative, with the fractional area of a peak being equal to the fraction of carbon atoms in the chemical environment indicated by the peak’s chemical shift, Raman and infrared spectroscopy cannot determine the concentrations of the structures detected, due to the dramatic influence of transition and scattering matrix elements on the band intensities. Whether a functional group is present at 2% or 10% concentration makes a big difference in the structural interpretation [17].

Carbon X-ray photoelectron spectroscopy (XPS) often shows only one or two bands while ^13^C NMR resolves at least six [18]. While aggressive deconvolution is typically used in XPS to generate more bands, it must be recognized that such deconvolution could also be applied to each of the resolved NMR bands. In a recent study comparing NMR and XPS of carbon overlayers made from glucose at 300 °C [18], the NMR spectrum could be reasonably deconvolved into 3 C=O (alkyl-/alkyl-, alkyl-/aromatic-, and aromatic-/aromatic-bonded), 2 COO (alkyl- vs. aromatic-bonded), 2 aromatic C–O (phenol vs. furan), 4 other aromatic peaks (CH, C not bonded to H, CH two bonds from O, and C not bonded to H and two bonds from O), 3 alkyl C–O (C–O–CH, HO–CH, and OCH_2_), 1 nonpolar CH_2_, 1 nonpolar CH, and 1 CH_3_ band, with evidence for these peaks from spectral editing and 2D NMR spectra [18]. So there would be a total of 17 deconvolved bands in NMR, compared to 4 in XPS. In the same study, deconvolution of unresolved XPS bands of carbon overlayers made at 600 °C led to an incorrect result: XPS suggested a significant alkyl fraction, while NMR, which fully resolves alkyl from other signals, categorically ruled this out [18]. Furthermore, XPS does not measure the composition of the bulk of the material; limited penetration depth is also a shortcoming of electron energy loss spectroscopy (EELS), in addition to sample degradation [19]. The interpretation of X-ray absorption near-edge spectroscopy (XANES) “is complicated by the fact that there is not a simple analytic (or even physical) description of XANES” [20]. Since “there is not a useful ‘XANES Equation’” [20], the XANES peak positions and intensities have no simple theoretical basis, while in ^13^C NMR the signal frequency is strictly proportional to the magnetic field at the carbon nucleus and the fractional peak area is the fraction of carbon atoms in a specific chemical environment.

NMR spectroscopy has been used in detailed, quantitative studies on the structure of amorphous carbon overlayers, derived from ^13^C-enriched sugars, in catalysts and catalyst supports [7,10]. ^13^C NMR spectroscopy can detect all carbon components, including C=O and C–O groups as well as sp^3^-hybridized carbons [7,11,12], even without isotopic enrichment [13], and provide an estimate of aromatic cluster size [13]. In addition, it can reveal molecular mobility [21,22]. In this work, we investigate the structure of more aromatic carbon overlayers prepared using an aromatic precursor, 2,3-naphthalenediol, on γ-Al_2_O_3_ and SiO_2_ supports, after heat treatments at 400 °C and 800 °C. 2,3-napthalenediol is nontoxic and inexpensive, and there are two advantages to using this precursor [23,24] instead of sucrose, which was used in our previous work [7,10]. First, pyrolysis of 2,3-napthalenediol at high temperatures leads to the formation of graphitic carbon, which is more stable under harsher hydrothermal conditions. Second, because 2,3-napthalenediol is not water soluble, it may be beneficial for carbon coating of oxide materials that cannot be exposed to water since they would hydrolyze. Our experiments take advantage of multiple cross polarization (multiCP, with built-in direct polarization) [25] for generating the ^13^C magnetization, which makes all components observable, including molecules undergoing large-amplitude, liquid-like motions. Challenges arise from the relatively low volume fractions of the thin carbon overlayers dispersed on the surface of mesoporous oxide supports, which limit the NMR signal strength, and from sample conductivity, which results in radio-frequency power absorption. Herein, we demonstrate that samples diluted in a superior nonconductive and carbon-free filler, beta-tricalcium phosphate (β-TCP), yield sufficient signal intensity for meaningful structural analysis. The use of multiCP allows for obtaining nearly quantitative ^13^C spectra within a reasonable measuring time, while direct polarization (DP) with a short recycle delay highlights the signals from carbon near unpaired electrons. Combination of multiCP or DP with dipolar dephasing or shift-anisotropy filtering is feasible and further reveals molecular mobility or paramagnetic shift anisotropy due to unpaired electrons.

## 2. Materials and Methods

### 2.1. Materials

2,3-Naphthalenediol, Davisil Grade 643 silica, and tricalcium phosphate (β-TCP) were purchased from Sigma-Aldrich (St. Louis, MO, USA). Alumina (γ-Al_2_O_3_) was purchased from Degussa (Zürich, Switzerland), acetone from Macron Fine Chemicals (Phillipsburg, NJ, USA), and polydimethylsilane (PDMS) from Scientific Polymer Products, Inc. (Ontario, NY, USA).

### 2.2. Preparation of Carbon Overlayers on Mesoporous Oxides

To coat the surface of silica or alumina with 20 wt % of an aromatic-rich carbon layer, a solution of 2,3-napthalenediol in acetone was added to the oxide support, and the mixture was stirred vigorously at room temperature until the acetone evaporated. Half a gram of the dried sample was loaded in a ceramic boat and placed in a quartz tube (20 mm ID). The tube was placed in a furnace and pyrolyzed under flowing N_2_ gas (90 SCCM) at 400 °C or 800 °C (5 °C·min^−1^ ramp) for 4 h. The sample name, e.g., NaphOH-SiO_2_-800, indicates the oxide support (‘SiO_2_’) and the pyrolysis temperature in °C (‘800’).

### 2.3. Tricalcium Phosphate Filler for NMR

Radio-frequency power absorption presents a challenge when performing NMR experiments on conductive materials such as carbon materials made by pyrolysis at high temperatures. It results in reduced detection efficiency, sample heating, potential arcing, and, even more importantly, altered pulse flip angles, degraded cross-polarization conditions, and lower-power ^1^H decoupling. These problems can be greatly reduced by diluting the conductive sample in nonconductive filler, which most importantly breaks the paths of eddy currents that are induced in the sample by the oscillating B_1_ field of the radio-frequency coil. Synthetic laponite clay, a nonconductive filler used in previous experiments, was found to produce alkyl and carbonate background signals after overnight signal averaging; see Figure S1a. Therefore, we searched for an alternative filler that gives no detectable carbon background signal and is minimally hygroscopic. We identified beta-tricalcium phosphate (β-TCP) as a superior replacement of laponite, without carbon background signals; see Figure S1b. β-TCP consists of soft, thin flakes that coat the conductive sample particles well and allow us to increase the sample loading, and therefore the NMR signal, nearly four-fold compared to laponite clay. 

Before use, the β-TCP powder was placed in a round-bottom flask, heated over a Bunsen burner for 30 min to remove moisture and organic carbon, and after cooling mixed with polydimethylsilane (PDMS) powder at a ~99:1 weight ratio. PDMS serves as an internal reference with a single carbon resonance at −3 ppm, outside of the common range of chemical shifts in carbon materials. With PDMS in the sample, null spectra from the material studied, with just the PDMS resonance, can be reliably distinguished from absence of signal due to potential spectrometer malfunction. The filler with PDMS was stored in a vacuum desiccator prior to mixing with the conductive sample.

Sample materials were ground and mixed with laponite or β-TCP filler in an agate mortar before being packed into a 4-mm rotor (over a 3-mm high glass insert). The weight ratio of sample to filler ranged from 35:65 to 47:53 depending on the sample conductivity, and the mass of sample packed in 4-mm rotors ranged from 28 to 42 mg. Specific values are listed in the Supplementary Material. Fillers with PDMS (109 mg of laponite-PDMS and 136 mg of TCP-PDMS) were also packed into rotors to examine whether these fillers introduce extraneous signals into the multiCP spectra.

### 2.4. Solid-State NMR Methods

The NMR experiments were performed on a Bruker Avance 400 spectrometer at a 100 MHz ^13^C resonance frequency using a Bruker double-resonance magic-angle spinning probehead for 4-mm rotors. All ^13^C spectra were measured at a spinning frequency of 14 kHz.

Quantitative ^13^C NMR spectra were obtained by the multiCP pulse sequence as described previously and by improved, composite-pulse multiCP [25]. The multiCP experiments used a recycle delay of 0.6 s, six 1.1 ms and one 0.55 ms 90%–100% ramp CP periods, and a 0.15-s delay for ^1^H repolarization. The modified multiCP pulse sequence replaced the direct 90° pulse in the conventional multiCP pulse sequence with nonorthogonal-phase 90°–180° pulses to reduce ^13^C magnetization loss due to pulse imperfections [25]. After a recycle delay of 0.45 s, the ramp for CP (86%–100%) on the ^1^H channel was implemented with eleven 1.1 ms and one 0.55 ms ramp CP periods, and a 0.1-s delay for ^1^H relaxation and repolarization. Nonprotonated and mobile fractions were identified by applying a period of recoupled dipolar decoupling of 68 μs to dephase magnetization of carbons with strong ^1^H dipolar coupling, such as in immobile CH and CH_2_ groups [26]. The total measuring time for a pair of multiCP and multiCP with recoupled dipolar decoupling experiments was 36 h per sample. The ^13^C spectra of NaphOH-SiO_2_-800 obtained with the conventional and modified multiCP sequences (Figure S2) are nearly identical and indicate good spectrometer reproducibility and the robustness of the multiCP methods.

A standard ramp CP experiment was applied to NaphOH-Al_2_O_3_-800 to selectively enhance signals of sp^3^-hybridized carbons. An 86%–100% amplitude ramp was applied on the ^1^H channel with a contact time of 1.1 ms and a recycle delay of 0.45 s. The measuring time for the ramp CP spectrum was 46 h. A direct polarization/magic angle spinning (DP/MAS) NMR spectrum with a short recycle delay of 1 s and its corresponding DP spectrum of mobile and nonprotonated segments selected by recoupled dipolar dephasing before detection were also recorded. The measuring time for each DP spectrum was ~46 h.

The five-pulse ^13^C chemical-shift-anisotropy (CSA) filter technique [27] used a CSA filtering time of 2*t*_r_ (4 × 31-μs dephasing time plus three 180° pulses) after the ^13^C excitation pulse, and was combined with a short (10-μs) gated decoupling period before detection to reduce signals from carbon sites with relatively large chemical or paramagnetic shift anisotropy or strongly coupled to ^1^H. With minimal (0.5-μs) dephasing time, this pulse sequence was also used for suppression of background signals from carbon-containing material in the probehead. The pulse flip angles outside the radio-frequency coil were far smaller than in the rotor and therefore the signal from outside the coil was not refocused; in particular, EXORCYCLE phase cycling [28] of the second 180° pulse and the 180° pulse for the Hahn echo before detection [29] reduced the background signal below the detection limit. 

The multiCP sequence was combined with a 3-s ^13^C *T*_1_ filter, which allows species with short *T*_1C_ to relax before detection and retains the carbon components with little large-amplitude mobility in NaphOH-SiO_2_-400. The five-pulse ^13^C CSA filter described above was applied after multiCP to suppress signals from immobilized, six-membered aromatic rings with relatively large CSA and select carbons with small shift anisotropy, e.g., due to near-isotropic mobility.

^1^H NMR spectra of NaphOH-SiO_2_-400 and NaphOH-Al_2_O_3_-400 were recorded at a spinning frequency of 7 kHz to detect mobility of molecules in the organic layers. The probehead background ^1^H NMR signal was suppressed by a simple and reliable one-pulse scheme described elsewhere [30]. In short, two one-pulse spectra, with pulse flip angles of β and 2β (e.g., 90° and 180°), were acquired. The essentially background-free spectrum was obtained by subtracting the second spectrum, scaled by 0.53 (=1/2 + 0.03 nonlinearity corrections), from the first. Chemical shifts were referenced to the ^1^H peak of hydroxyapatite (standard material from NIST, Gaithersburg, MD, USA) at 0.18 ppm.

## 3. Results

**Figure 1:**
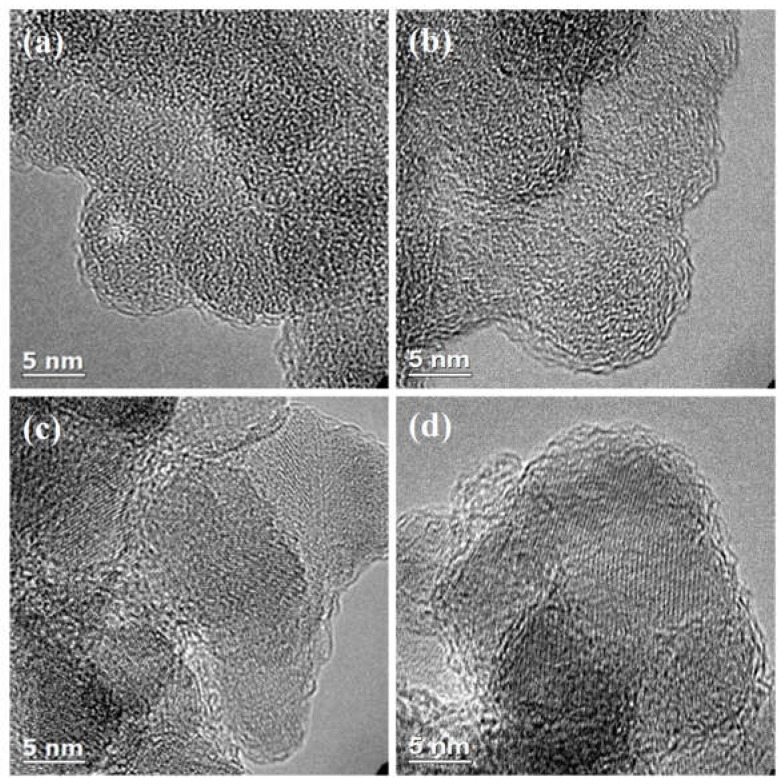
High-resolution transmission electron microscopy (HRTEM) images of 2,3-naphthalenediol heat-treated at 400 °C on (**a**) SiO_2_ and (**c**) Al_2_O_3_, and heat-treated at 800 °C on (**b**) SiO_2_ and (**d**) Al_2_O_3_.

Figure 1 presents HRTEM images of NaphOH-SiO_2_-400, NaphOH-SiO_2_-800, NaphOH-Al_2_O_3_-400, and NaphOH-Al_2_O_3_-800, all of which show thin carbon overlayers on the surfaces of silica or alumina. Even though we can clearly observe the presence of carbon after coating, it is difficult to distinguish between amorphous carbon [10] and graphitic carbon overlayers by HRTEM, or to determine the extent of carbonization in the pyrolyzed samples; solid-state NMR can address these questions [13].

**Figure 2:**
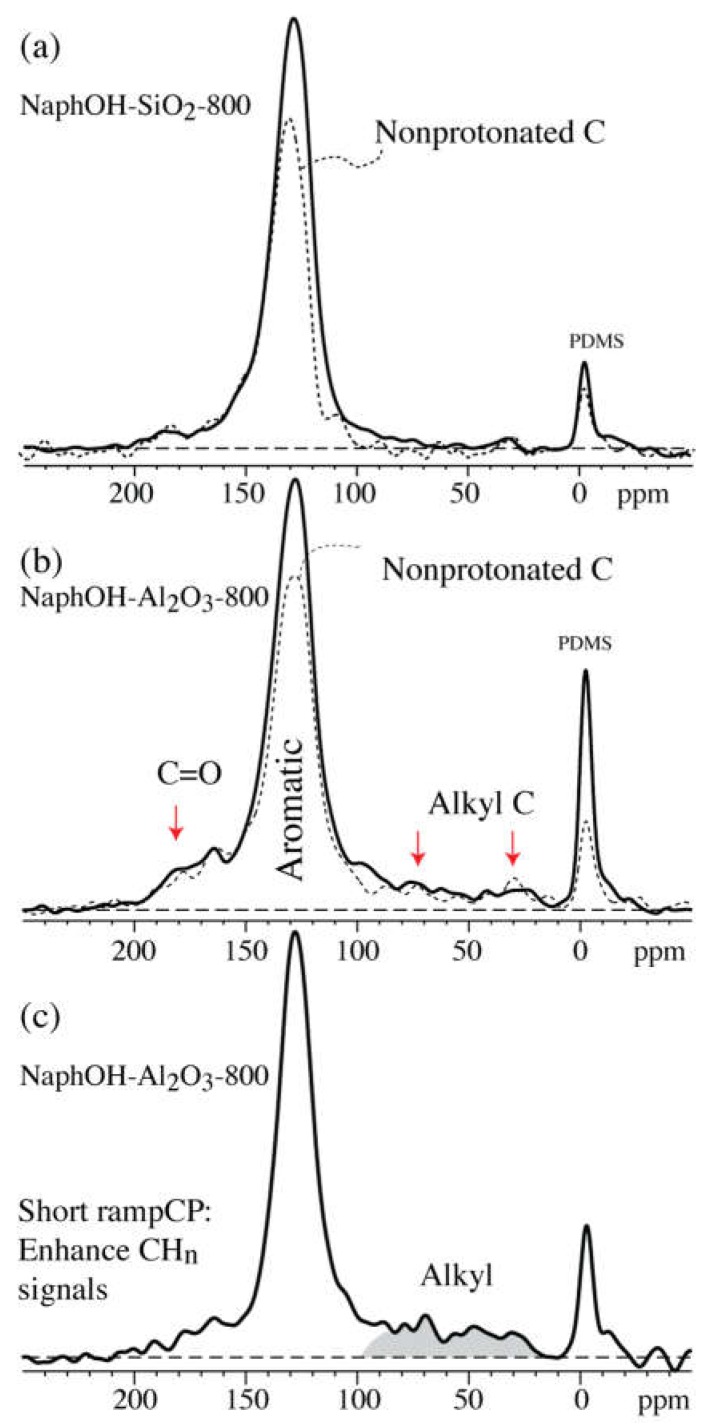
Nearly quantitative multiCP ^13^C NMR spectra of 2,3-naphthalenediol heat-treated at 800 °C on (**a**) SiO_2_ and (**b**) Al_2_O_3_. The corresponding spectra of nonprotonated C and mobile segments after 68-µs dipolar dephasing are shown as dashed lines. (**c**) ^13^C signals of sp^3^-hybridized carbons (shaded area) on Al_2_O_3_ after 1.1-ms ramp cross polarization from ^1^H.

Figure 2 shows the multiCP ^13^C NMR spectra of carbon overlayers formed on the SiO_2_ and γ-Al_2_O_3_ supports by pyrolysis at 800 °C (NaphOH-SiO_2_-800 and NaphOH-Al_2_O_3_-800), with their corresponding spectra of the nonprotonated carbon atoms (and mobile segments) superimposed. The dominant peak in spectra of both samples is from sp^2^-hybridized, C=C carbons, most of which are not protonated. The spectra of NaphOH-Al_2_O_3_-800 also exhibit significant nonaromatic components (Figure 2b). To assure the reproducibility of these results, we performed the same NMR experiments on a second sample (see Figure S2), which confirmed the nonaromatic species in NaphOH-Al_2_O_3_-800. The presence of sp^3^-hybridized carbons is also documented in a spectrum acquired after short ramp CP from ^1^H, which selectively enhances the signals from CH_n_ relative to C=C carbons (Figure 2c).

**Figure 3:**
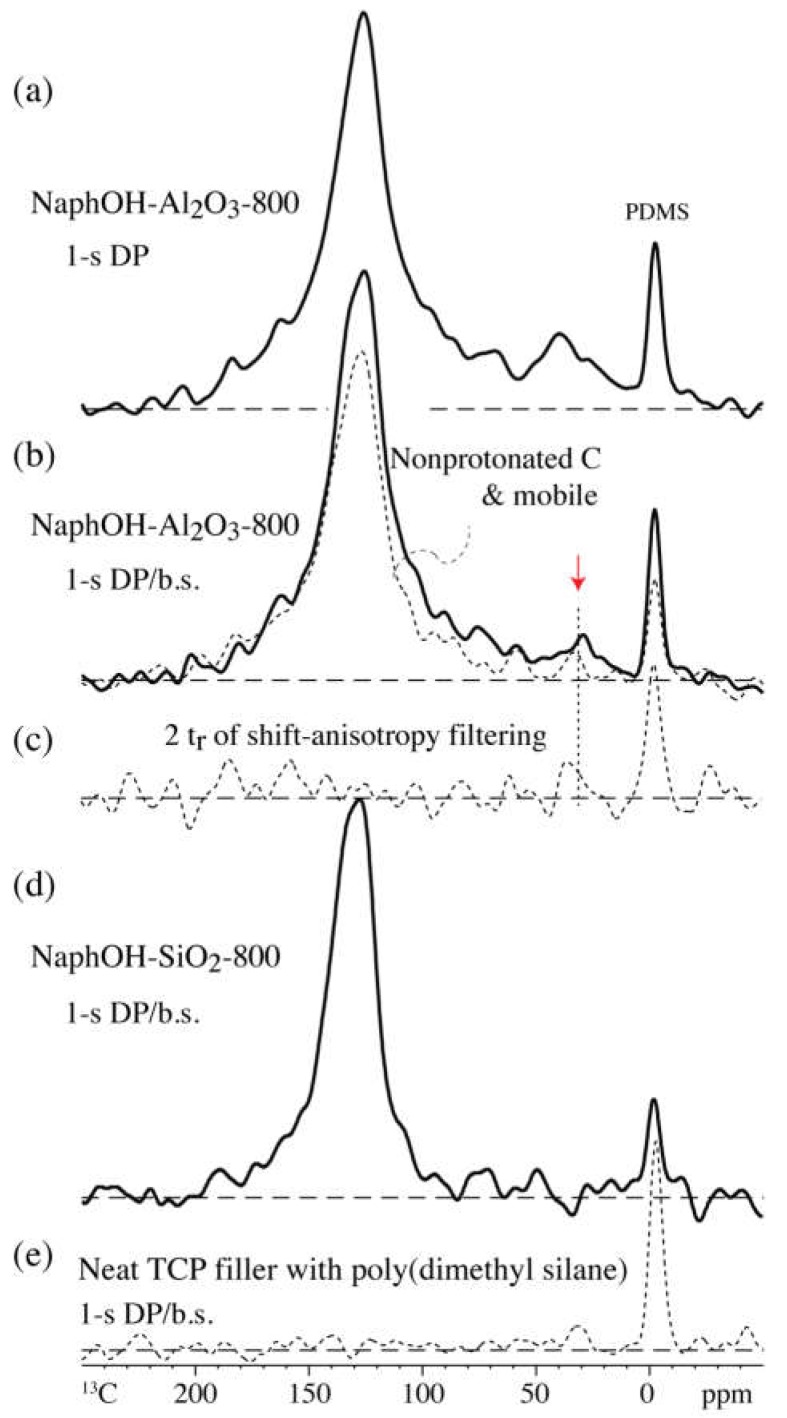
^13^C spectra of 2,3-naphthalenediol heat-treated at 800 °C after 1-s direct polarization (solid lines). (**a**) On Al_2_O_3_; (**b**) on Al_2_O_3_ with probe-head background suppression (b.s.); the spectrum after dipolar dephasing (dashed line) is also shown; the signal of sp^3^-hybridized carbon near 35 ppm is highlighted by a red arrow; (**c**) same as (**b**) after 4 × 31 μs shift-anisotropy recoupling. (**d**) On SiO_2_ with probe-head background suppression. (**e**) For reference, spectrum of neat TCP filler with PDMS after probe-head background suppression.

The ^13^C direct-polarization spectra of NaphOH-Al_2_O_3_-800 with a short recycle delay (1 s) are presented in Figure 3a,b, without and with probe-head background suppression, respectively. They show the broad signals of the sp^3^-hybridized carbon more prominently, and in particular, a peak near 32 ppm. In the corresponding spectrum after dipolar dephasing (Figure 3b, dashed line), the signals of a large fraction of these sp^3^-hybridized carbons are retained, indicating that they are not protonated. Moderate shift-anisotropy recoupling, which leaves the PDMS signal nearly unaffected, strongly dephases most other signals, including those of the sp^3^-hybridized carbons resonating between 10 and 70 ppm (Figure 3c); this is unusual, indicating the effect of paramagnetic shift anisotropy generated by unpaired electrons due to the dipolar coupling between the thermally averaged electron magnetic dipole moment and the nuclear spin [31,32,33,34,35,36]. Spinning sidebands and shift anisotropy dephasing due to paramagnetic shift anisotropy in detonation nanodiamond are documented in Figure S3.

**Figure 4:**
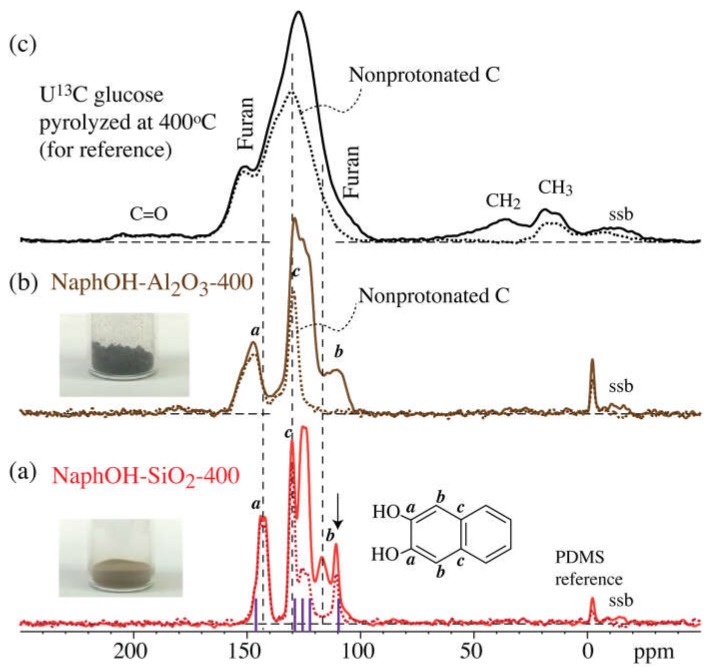
MultiCP ^13^C NMR spectra (solid lines: full spectra; dashed lines: after dipolar dephasing) after heat treatment at 400 °C. (**a**,**b**): 2,3-naphthalenediol heat-treated on (**a**) SiO_2_ and (**b**) Al_2_O_3_. “ssb”: spinning sideband of the aromatic peak. The stick spectrum (purple vertical lines) in (**a**) marks the resonance positions of neat 2,3-naphthalenediol. (**c**) Bulk glucose char after 400 °C pyrolysis, for reference.

The multiCP ^13^C NMR spectra of the carbon overlayers formed on the Al_2_O_3_ and SiO_2_ supports at 400 °C (NaphOH-Al_2_O_3_-400 and NaphOH-SiO_2_-400) are displayed in Figure 4. These spectra show signals exclusively from C=C carbons. The absence of sp^3^-hybridized carbon and C=O signals rules out the presence of residual solvent in 400 °C samples, therefore indicating that the sp^3^-hybridized and C=O carbons observed in NaphOH-Al_2_O_3_-800 were not derived from residual solvent. The characteristic peaks from 2,3-naphthalenediol (see vertical dashed lines) are recognizable in the spectra of the 400 °C samples, but some peak maxima have shifted by >5 ppm.

**Figure 5:**
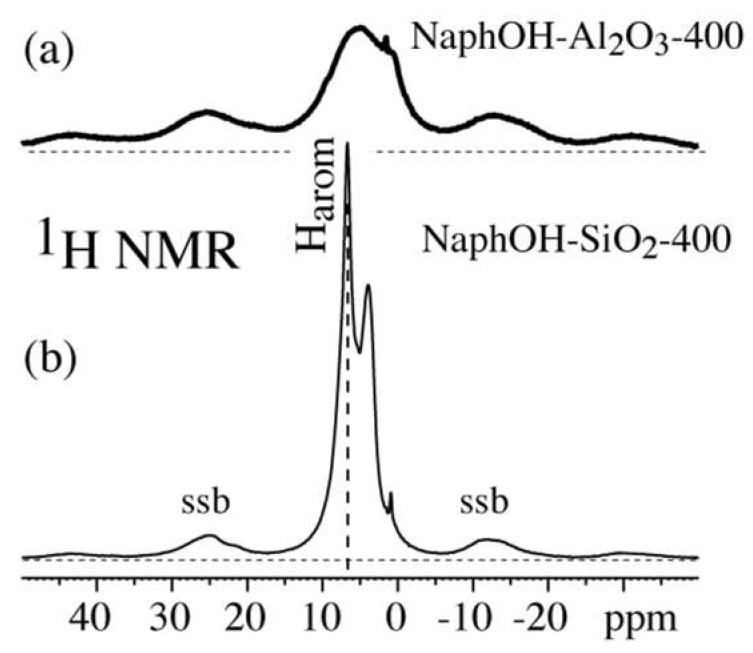
^1^H NMR spectra of 2,3-naphthalenediol heat-treated at 400 °C on (**a**) Al_2_O_3_ and (**b**) SiO_2_.

**Figure 6:**
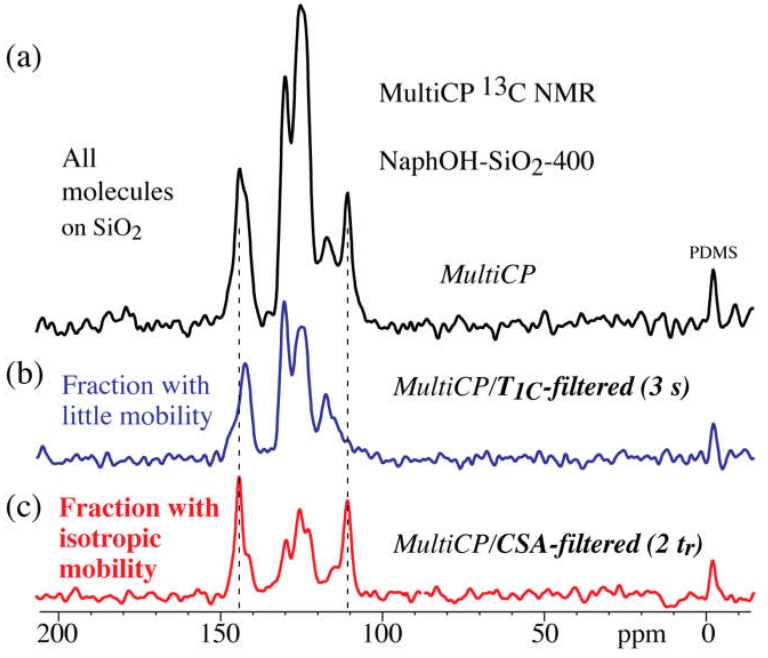
Selective ^13^C NMR spectra of 2,3-naphthalenediol heat-treated at 400 °C on SiO_2_. (**a**) Reference spectrum of all carbon after nominal 1-ms *T*_1C_ filter. (**b**) Spectrum after 3-s *T*_1C_ filter. (**c**) Spectrum after 4 × 31-µs chemical-shift anisotropy (CSA) filter.

Notably, both ^13^C and ^1^H spectra (Figure 4a and Figure 5b) of NaphOH-SiO_2_-400 show additional sharp peaks. Their assignment was assisted by selective ^13^C NMR experiments such as ^13^C *T*_1_ and CSA filtering (Figure 6b,c, respectively). The spectrum after a 3-s ^13^C *T*_1_ filter selectively retains the broader signals of carbon segments with low mobility (specifically, with small spectral density of fluctuating magnetic fields at 2π 100 MHz). The sharp peaks that have been suppressed here are retained after CSA filtering, which removes signals of C=C carbons with little or no mobility [27]. The chemical shifts, slow chemical-shift anisotropy dephasing, narrow lines, and fast *T*_1C_ relaxation indicate that the sharp signals from NaphOH-SiO_2_-400 are due to unreacted 2,3-naphthalenediol with fast, nearly isotropic mobility.

## 4. Discussion

### 4.1. Comparison of Carbon Overlayers on Al_2_O_3_ vs. SiO_2_ at 800 °C

The spectra of the carbon overlayer on SiO_2_ show mostly C=C signals, which we assign mostly to aromatic C. Signals at 130 ppm could in principle arise from alkenes rather than aromatic rings. However, there are many reasons against a dominant alkene fraction: Alkenes are not sufficiently conductive to explain the high conductivity observed in terms of the significantly broadened electronic resonance of the probe head at the ^1^H NMR frequency. Since the starting material in our study consists already of two fused aromatic rings, comparisons with amorphous carbon made from nonaromatic precursors, e.g., by chemical vapor deposition [37,38], are inapplicable. Due to aromatic stabilization, converting the fused aromatic rings into alkenes would be unfavorable not only kinetically but also thermodynamically. Furthermore, NMR has shown that a majority of the C=C (sp^2^-hybridized) carbons are not bonded to hydrogen; a nearly pure alkene network with little hydrogen substitution and 120° bond angles would almost unavoidably produce six-membered rings that would end up becoming aromatic. 

The carbon overlayers on Al_2_O_3_ reproducibly also exhibit significant nonaromatic components. A pronounced shoulder extending from 180 ppm to 160 ppm can be attributed to various types of C=O moieties, while a broad “foot” extending from 70 to 15 ppm is assigned to sp^3^-hybridized C. The latter signal is enhanced, relative to the aromatic-carbon peak, by ramp-CP from ^1^H (Figure 2c) and by direct polarization with short 1-s recycle delay (Figure 3a,b).

### 4.2. Tetrahedral Carbon Species Formed on the Al_2_O_3_ Support at 800 °C

The observed formation of sp^3^-hybridized carbons from a purely aromatic precursor was unexpected; this effect has not been observed in carbon materials made by pyrolysis of carbohydrates (primarily glucose) [7]. It is reassuring to note that conversion of aromatic to sp^3^-hybridized carbon can be thermodynamically favorable [39]. The sp^3^-hybridized carbon signals cannot be attributed to residues from the solvent, acetone, used in the deposition of naphthalenediol on the Al_2_O_3_ support, because the spectra after heat treatment at 400 °C (Figure 4b) show no detectable signals of the CH_3_ groups of acetone or, in fact, any alkyl segments.

Chemical shifts of carbon bonded to four other carbon atoms (i.e., quaternary carbons) range from 30 to 70 ppm. Relatively sharp resonances between 30 and 40 ppm are characteristic of diamond: bulk and microdiamond typically resonates between 35 and 39 ppm, while nanodiamond, in addition to the main signal at 35 ppm, exhibits a broad foot extending to ~60 ppm [40,41]. In highly branched alkanes, chemical-shift increment rules [42,43] predict about 55 ppm for quaternary carbons with the maximum number of β- and γ-carbons. Inhomogeneously broadened bands around ~68 ppm are typical of the tetrahedrally bonded atoms in amorphous carbon films [44,45,46].

Analysis of the couplings of ^13^C in the sp^3^-hybridized sites, in particular those resonating near 32 ppm, on the Al_2_O_3_ support reveals unusual behavior characteristic of a defect-rich diamond lattice. The nonprotonated sp^3^-hybridized carbon sites on Al_2_O_3_ exhibit very short ^13^C spin–lattice (*T*_1C_) relaxation times, which makes it possible to observe them relatively selectively and efficiently by direct polarization with short 1-s recycle delay (Figure 3b). Similarly, Alam [47] identified some “diamond-like” carbon species with relatively short ^13^C spin-lattice relaxation (*T*_1C_) times in synthetically produced thin diamond layers. This fast relaxation is an indication of fluctuating fields of unpaired electrons. This effect has been documented in detail in detonation nanodiamond, where between 10 and 30 unpaired electrons per 10,000 carbons [48,49] shortened *T*_1C_ to a few seconds, producing a strongly nonexponential relaxation behavior [41,50,51] due to the pronounced dependence of the relaxation time on the electron-carbon distance [52]. Fluctuating fields of ^1^H nuclear spins cannot be the origin of the short *T*_1C_, since dipolar dephasing (see Figure 3b, dashed line) shows that most of the sp^3^-hybridized carbon atoms are not bonded to hydrogen. The observed absence of strong dipolar C–H couplings cannot be attributed to motional averaging, since the sp^3^ carbons also show large shift anisotropies, which would be averaged together with the dipolar couplings. In fact, the shift anisotropies are significantly larger than typical chemical-shift anisotropies of sp^3^-hybridized C (Figure 3c); under the same conditions, the CH_2_ and CH_3_ groups in a glucose char show little signal loss (Figure S4).

The unusually large shift anisotropies of the sp^3^-hybridized carbon must again be attributed to unpaired electrons, in this case to their average dipolar fields. The resulting paramagnetic shift anisotropy (PSA) [31,32,33,34,35,36] has very similar effects as chemical-shift anisotropies (CSAs), but in our present sample they are larger than CSAs of sp^3^-hybridized carbons, which are small to moderate due to the nearly tetrahedral bonding environment of these carbons. In detonation nanodiamond, PSA gives rise to inhomogeneous line-broadening in static samples and to spinning sidebands under MAS (see Figure S3). It is likely that as in nanodiamond, the unpaired electrons of the carbon overlayers can be attributed to dangling bonds in a defective diamond lattice of the sp^3^-hybridized carbons.

### 4.3. Structure after 400 °C Heat Treatment

Heat treatment of 2,3-naphthalenediol at 400 °C on the SiO_2_ and Al_2_O_3_ supports resulted in yellow-brown and nearly black samples, respectively; see Figure 4a,b. The darker color might suggest more extensive carbonization on Al_2_O_3_. However, both spectra show almost exclusively peaks characteristic of 2,3-naphthalenediol. In addition, the predominance of protonated aromatic C revealed by dipolar dephasing is typical of individual 2,3-naphthalenediol molecules and unlike the hydrogen-poor, more condensed or interlinked aromatic structures of chars, including the 400 °C glucose char shown for reference in Figure 4c. There are no indications of additional nonprotonated aromatic carbons with C–C bonds to other 2,3-naphthalenediol molecules, which would produce additional intensity, if not new peaks, in the dipolar-dephased spectrum. Therefore, any linkage of napthalenediol molecules would have to be through ether oxygen, connecting to another napthalenediol molecule or to the oxide substrate. Significant interactions through the C–O moieties are indeed indicated by the substantial chemical shift changes, in particular on SiO_2_, by up to 5 ppm observed for the aromatic C–O and their neighboring carbons (resonating around 145 and 110 ppm, respectively). The fact that these changes differ by several ppm between the two supports suggests that these effects are due to the substrate rather than other naphthalenediol molecules. This is additional evidence of distinctly different interactions of alumina and silica with the naphthalenediol overlayer.

### 4.4. Highly Mobile Naphthalenediol on SiO_2_

The ^1^H NMR spectrum of 400 °C heat-treated 2,3-naphthalenediol on SiO_2_ (Figure 5b) shows some sharp signals indicative of highly mobile molecules. The CSA-filtered ^13^C NMR spectrum (see Figure 6c) confirms that a significant fraction of 2,3-naphthalenediol undergoes large-amplitude, nearly liquid-like motions. The positions of the sharp peaks of the mobile molecules match those of neat 2,3-naphthalenediol (i.e., 146 ppm for C_a_, 110 ppm for C_b_, 130 ppm for C_c_, Figure 4a), which indicates that these mobile molecules interact mostly with other naphthalenediols. We hypothesize that these 2,3-naphthalenediol molecules move freely on a layer of naphthalenediol that is bound to the curved pore surface; due to the curvature, motion along the surface leads to nearly isotropic dynamic averaging of anisotropic NMR interactions. Similar large-amplitude mobility is observed for glucose molecules on mesoporous SiO_2_; see Figure S5. Based on the relative peak intensities in Figure 4, Figure 5 and Figure 6, we estimate that at a minimum, 1/3 of the molecules are undergoing fast, nearly isotropic motions.

## 5. Conclusions

Solid-state NMR characterization of 2,3-naphthalenediol pyrolyzed or heat-treated on Al_2_O_3_ and SiO_2_ has revealed several unexpected structural and dynamical differences. Whereas the carbon film made at 800 °C on SiO_2_ is mostly aromatic in structure, additional oxygen-containing and sp^3^-hybridized moieties are observed on Al_2_O_3_. While the formation of sp^3^-hybridized carbon fragments from a purely aromatic precursor may be surprising, the experimental evidence indicates that these sp^3^-hybridized carbons were not derived from residual solvent, according to spectra of the same material treated at 400 °C where no alkyl or ketone signals were observed. NMR showed low hydrogen and indications of high unpaired-electron concentrations near the sp^3^-hybridized carbons, which is strong evidence for a disordered diamandoid structure. Heat treatment at 400 °C did not produce extensive carbonization, but immobilization of naphthalenediol is observed on the Al_2_O_3_ substrate. On SiO_2_, about 2/3 of the naphthalenediol is strongly adsorbed and chemical-shift changes of the C–O carbon indicate strong interaction with the substrate, but at least 1/3 of the molecules are undergoing large-amplitude motions.

## Supplementary Materials

The following are available online at http://www.mdpi.com/1996-1944/11/6/980/s1, Figure S1: MultiCP ^13^C NMR spectra of neat filler and PDMS, Figure S2: Tests of the reproducibility of sample preparation and multiCP ^13^C NMR, Figure S3: Paramagnetic shift anisotropy effects in nanodiamond, Figure S4: Typical chemical-shift anisotropy (CSA) filtered multiCP ^13^C NMR, Figure S5: NMR of glucose on mesoporous silica.
