# Supplementary material for: Protective Carbon Overlayers from 2,3-Naphthalenediol Pyrolysis on Mesoporous SiO2 and Al2O3 Analyzed by Solid-State NMR
Source: Materials (Basel). 2018 Jun 9;11(6):980. doi: 10.3390/ma11060980 (PMC6025365; doi:10.3390/ma11060980)
Supplement: Supplementary file 1 [file materials-11-00980-s001.pdf]

Supplementary Material

for

Protective carbon overlayers from 2,3-naphthalenediol pyrolysis on mesoporous SiO_2 and Al_2O_3 analyzed by solid-state NMR

Pu Duan[1], Xiaoyan Cao[1], Hien Pham[2], Abhaya Datye[2], Klaus Schmidt-Rohr[1] *

[1] Department of Chemistry, Brandeis University, Waltham, MA 02453, USA

[2] Department of Chemical & Biological Engineering and Center for Microengineered Materials, University of New Mexico, Albuquerque, NM 87131, USA

* Correspondence: srohr@brandeis.edu; Tel.: (781) 736-2520

Specific inert filler concentrations of samples measured by NMR. A nonconductive filler was made from beta-tricalcium phosphate (β-TCP) with 1 wt% polydimethylsilane (PDMS) powder added as an internal NMR reference. NaphOH-Al_2O_3-800 (old) was mixed with TCP-PDMS at a 35:65 mass ratio, with 32.2-mg of sample in the rotor, while NaphOH-Al_2O_3-800 (new) was at a 42:58 mass ratio, with 36.4-mg of sample. For NaphOH-SiO_2-800 (old), the mass ratio was 46.7:53.3, with 30.5-mg of sample, while for NaphOH-SiO_2-800 (new), the mass ratio was 40:60, with 27.8-mg of sample. NaphOH-Al_2O_3-400 was mixed at 44.2:55.8, with 41.6-mg of sample, while NaphOH-SiO_2-400 was mixed at 44.6:55.4, with 32.2-mg of sample. The amounts of neat laponite-PDMS and of neat TCP-PDMS packed into 4-mm rotors were 109 mg and 136 mg, respectively. The PDMS signal varies between samples as a result of the different loadings of filler plus PDMS.

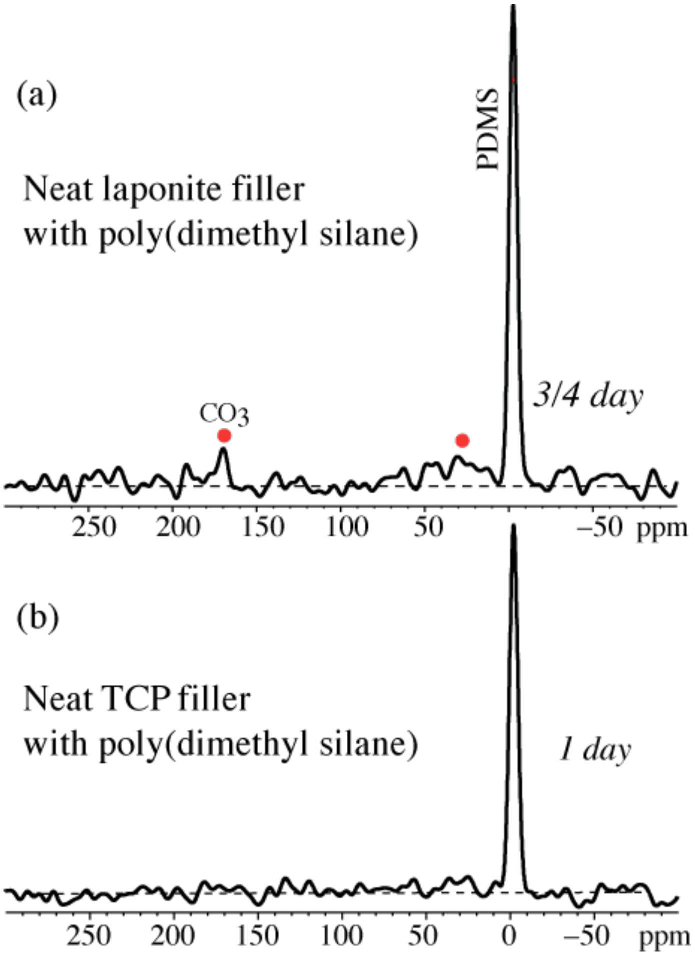

Figure S1. MultiCP ^{13}C NMR spectra of neat filler with PDMS. (a) Laponite clay + PDMS, 18-h signal averaging. Carbonate and alkyl signals are observed. (b) TCP + PDMS, 22-h signal averaging, without extraneous signals.

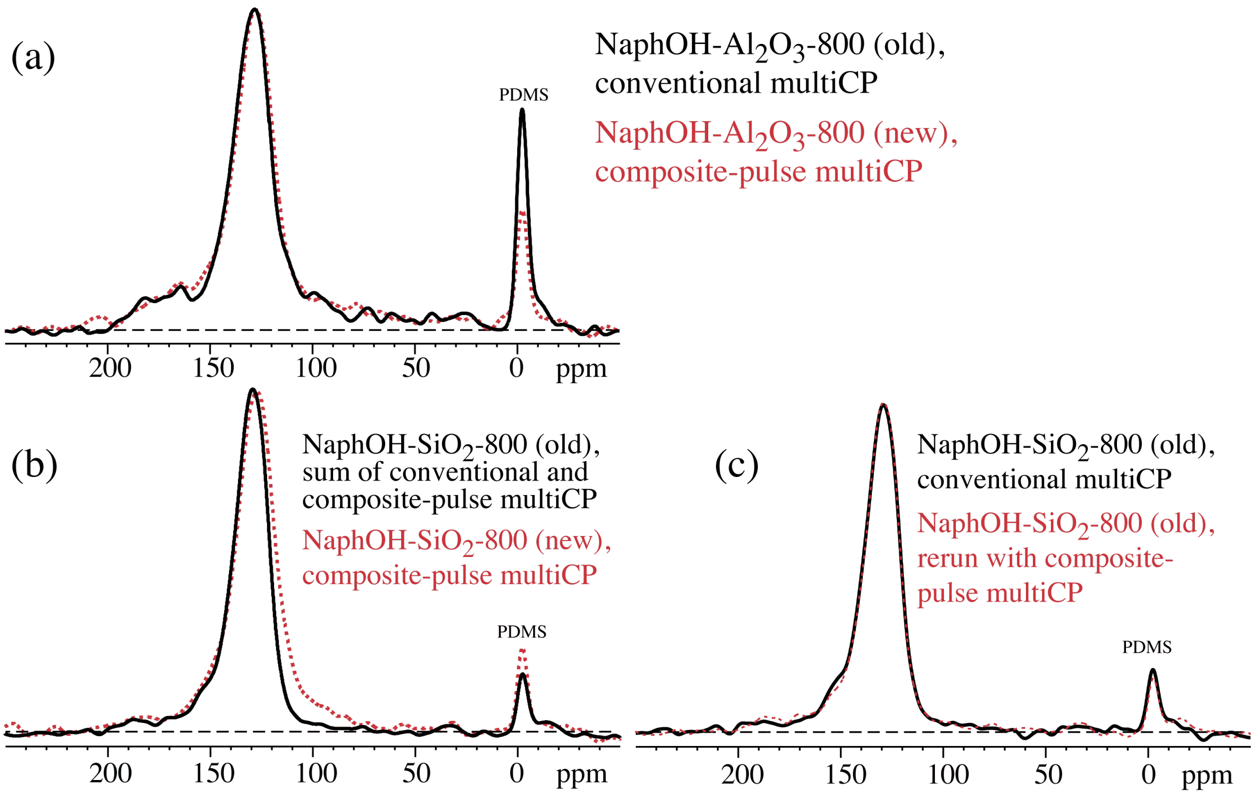

Figure S2. Tests of the reproducibility of sample preparation and multiCP ^{13}C NMR. (a) Old (solid black line) vs. new sample (red dashed line) of NaphOH-Al_2O_3-800. The match of the spectral patterns is excellent, confirming that the nonaromatic carbons in this material show up reproducibly and cannot be attributed to, for instance, a malfunction of the oven for thermal treatment. The variation of the PDMS signal is the result of different loadings of filler plus PDMS in the two samples. (b) Old (solid black line) vs. new sample (red dashed line) of NaphOH-SiO_2-800. The new material on SiO_2 has a wider aromatic signal, but it is still narrower than on Al_2O_3 and shows less intensity at < 70 ppm (alkyl C) and > 160 ppm (C=O). (c) The same material (old sample of NaphOH-SiO_2-800) measured five months apart. The spectra match without a scaling factor.

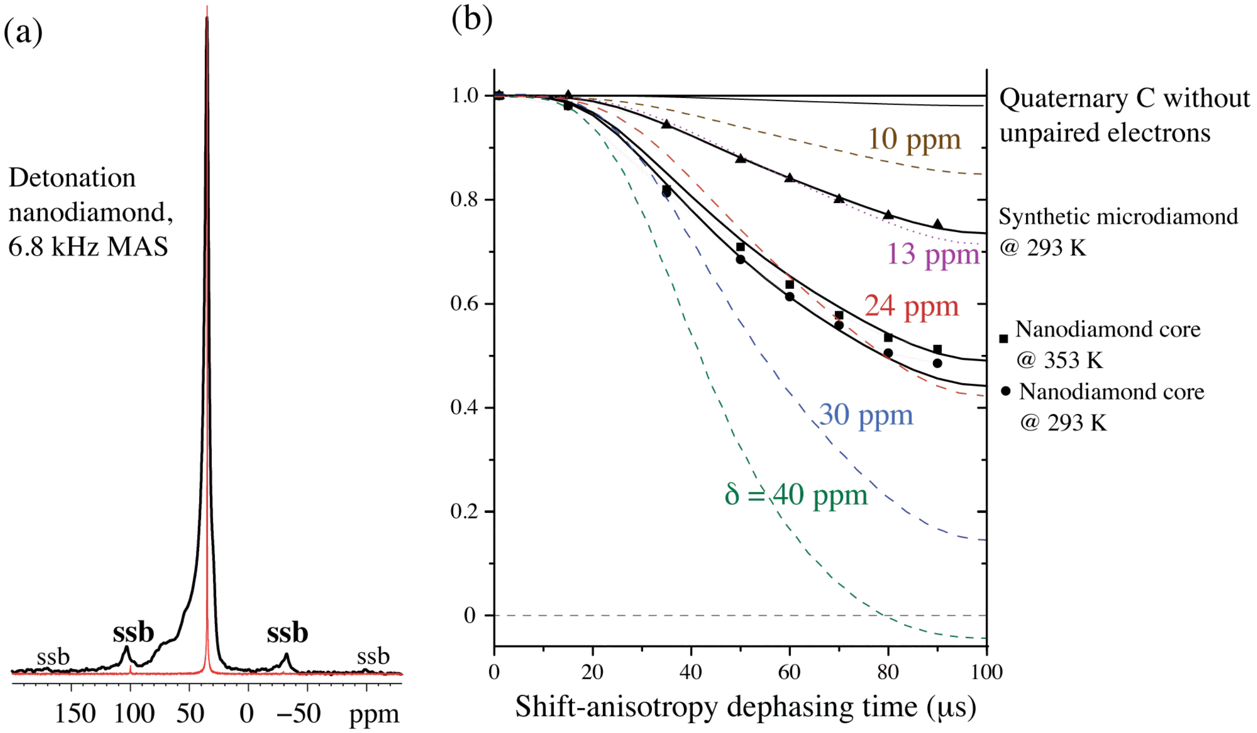

Figure S3. Evidence for paramagnetic shift anisotropy in pristine purified detonation nanodiamond with ~20 unpaired electrons per 10,000 C (10^{20} unpaired electrons per gram),[1-3] in a 9.4 T magnetic field. (a) Direct-polarization ^{13}C NMR spectrum of (thick line) air-oxidized detonation nanodiamond [4] and (thin read line) microdiamond recorded at 6.8 and 6.5 kHz MAS with 8 s and 100 s recycle delays, respectively. First- and second-order spinning sidebands characteristic of shift anisotropy are labeled "ssb". (b) Five-pulse recoupled chemical shift anisotropy dephasing at 5 kHz MAS [5] of (top) microdiamond and (bottom) the core of detonation nanodiamond from Sigma-Aldrich [6]. A slight decrease in the observed dephasing at 353 K is consistent with a decrease in the paramagnetic shift anisotropy due to thermal averaging of the electron spin (based on Curie's 1/*T* law). Dashed lines are simulated curves for chemical-shift anisotropy parameters between 10 and 40 ppm as indicated.

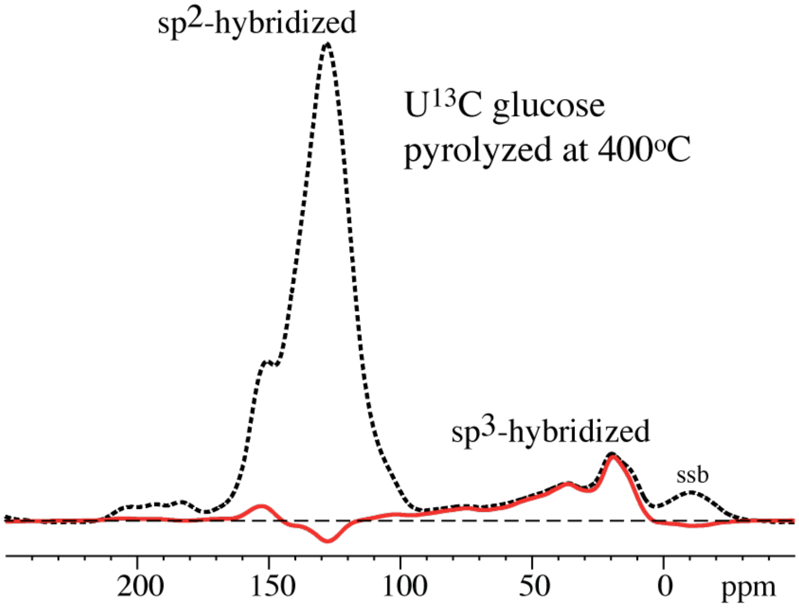

Figure S4. Typical chemical-shift anisotropy (CSA) filtered multiCP ^{13}C NMR spectrum of a low-temperature pyrolysis char. The dephasing of the signals of sp^3-hybridized carbons is minimal. Dashed line: Full spectrum after minimal CSA recoupling, for reference. Solid red line: Spectrum after ~2 $t_r = 4 \times 31$ μs of CSA recoupling.

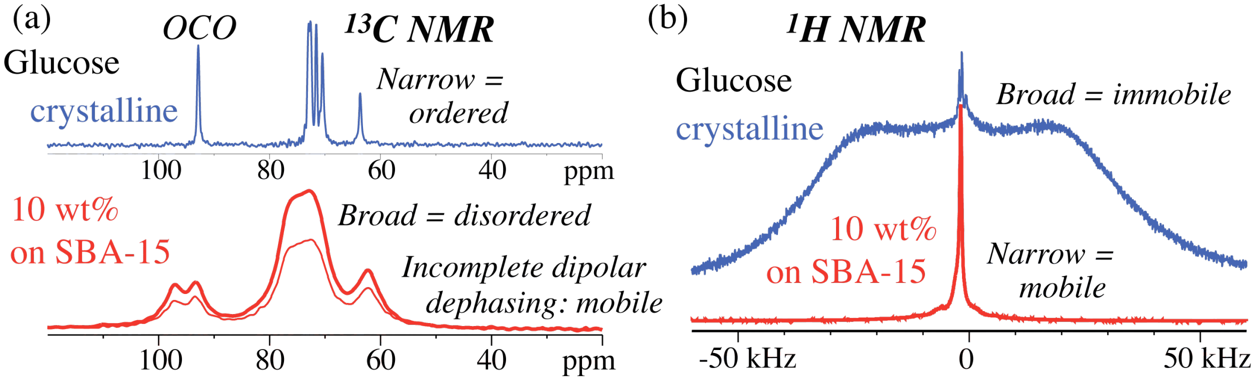

Figure S5. NMR of glucose on mesoporous silica. (a) ^{13}C and (b) ^{1}H spectra of (top) neat crystalline glucose and (bottom) $^{13}C_6$ glucose (10 wt%) deposited from aqueous solution onto mesoporous silica SBA-15. The glucose overlayer shows broad ^{13}C NMR bands, incomplete dipolar dephasing (thin red line), and ^{1}H motional line narrowing, which are characteristics of an amorphous layer with significant molecular mobility.

Additional References Cited

1. Shames, A. I.; Panich, A. M.; Kempinski, W.; Alexenskii, A. E.; Baidakova, M. V.; Dideikin, A. T.; Osipov, V. Y.; Siklitski, V. I.; Osawa, E.; Ozawa, M.; Vul, A. Y. Defects and impurities in nanodiamonds: EPR, NMR and TEM study. *J. Phys. Chem. Solids* **2002**, *63*, 1993-2001.
2. Levin, E. M.; Fang, X. W.; Bud'ko, S. L.; Straszheim, W. E.; McCallum, R. W.; Schmidt-Rohr, K. Magnetization and 13C NMR spin-lattice relaxation of nano-diamond powder. *Phys. Rev. B Condens. Matter Mater. Phys.* **2008**, *77*, 054418/1-054418/10.
3. Fang, X.; Mao, J.; Levin, E. M.; Schmidt-Rohr, K. Nonaromatic Core-Shell Structure of Nanodiamond from Solid-State NMR Spectroscopy. *J. Am. Chem. Soc.* **2009**, *131*, 1426-1435.
4. Osswald, S.; Yushin, G.; Mochalin, V.; Kucheyev, S. O.; Gogotsi, Y. Control of sp2/sp3 Carbon Ratio and Surface Chemistry of Nanodiamond Powders by Selective Oxidation in Air. *J. Am. Chem. Soc.* **2006**, *128*, 11635-11642.
5. Mao, J.-D.; Schmidt-Rohr, K. Separation of aromatic-carbon ^{13}C NMR signals from di-oxygenated alkyl bands by a chemical-shift-anisotropy filter. *Solid State NMR* **2004**, *26*, 36-45.
6. Fang, X. W.; Mao, J. D.; Levin, E. M.; Schmidt-Rohr, K. Nonaromatic core-shell structure of nanodiamond from solid-state NMR. *J. Am. Chem. Soc.* **2009**, *131*, 1426-1435.
